# Is DRE essential for the follow up of prostate cancer patients? A prospective audit of 194 patients

**DOI:** 10.1186/1471-2490-5-1

**Published:** 2005-01-10

**Authors:** Narasimhan Ragavan, Vijay K Sangar, Sujoy Gupta, Jennifer Herdman, Shyam S Matanhelia, Michael E Watson, Rosemary A Blades

**Affiliations:** 1 Department of Urology, Lancashire Teaching Hospitals NHS Trust, Preston, Lancashire, United Kingdom

## Abstract

**Background:**

Prostate cancer follow up forms a substantial part of the urology outpatient workload. Nurse led prostate cancer follow up clinics are becoming more common. Routine follow-up may involve performing DRE, which may require training.

**Objectives:**

The aim of this audit was to assess the factors that influenced the change in the management of prostate cancer patients during follow up. This would allow us to pave the way towards a protocol driven follow up clinic led by nurse specialists without formal training in DRE.

**Results:**

194 prostate cancer patients were seen over a period of two months and all the patients had DRE performed on at least one occasion. The management was changed in 47 patients. The most common factor influencing this change was PSA trend. A change in DRE findings influenced advancement of the clinic visit in 2 patients.

**Conclusions:**

PSA is the most common factor influencing change in the management of these patients. Nurse specialists can run prostate cancer follow-up clinics in parallel to existing consultant clinics and reserve DRE only for those patients who have a PSA change or have onset of new symptoms. However larger studies are required involving all the subgroups of patients to identify the subgroups of patients who will require DRE routinely.

## Introduction

Prostate cancer ranks first amongst all male urological cancers [1]. In the UK, 26027 new patients were diagnosed with prostate cancer during 2001 [1]. The evidence suggests an increasing trend in the incidence in the recent years, being 18201 in 1997 [2]. Nonetheless, better treatment modalities and earlier detection has resulted in a decrease in cancer related mortality [3]. This is shown in the age-standardized death rate per million population for prostate cancer, being 302 and 274 in 1991 and 2001 respectively.

Widespread PSA testing and increased awareness has led to the detection of early prostate cancer in many patients [4]. This has probably resulted in more patients requiring long periods of follow up. Nurse Specialists in UK health care system have evolved to share the increasing demand on the clinicians to meet the targets and waiting times in all the specialties. In urology, Nurse Specialists have assumed various roles including prostate assessment clinics, urodynamics and flexible cystoscopy [5]. In some health care trusts, Nurse Specialists are involved in the follow up of treated prostate cancer patients.

Faithfull et al studied the use of telephone follow up of prostate cancer patients by nurse specialists. They found that this method of follow-up at 3, 6 and 12 weeks post radiotherapy was effective and economical [6]. In addition a study on the follow-up of prostate cancer patients by on-demand contact with a nurse specialist was found to be as effective as traditional outpatient follow up by urologists [7].

The EAU guidelines [8] suggest that prostate cancer patients should be followed at regular intervals with a disease specific history and PSA estimation supplemented by digital rectal examination. This would suggest that all Nurse Specialists undertaking the role of follow-up of such patients should be trained in DRE. Data on the role of DRE in the follow up of prostate cancer patients is available only for the subgroup of patients who have had treatment with curative intent (radical prostatectomy or radical radiotherapy) and these studies show that PSA trend plays a more important role than DRE. However there is limited data available on the role of DRE and other factors (e.g. LUTS, Bone pain etc) in the follow up of diagnosed prostate cancer patients in the general setting involving all treatment varieties which is likely to be encountered in a nurse led follow up clinic.

The aim of this audit was to prospectively assess the various factors that influence a change in the management of the prostate cancer patients on follow up and to highlight the feasibility of nurse led clinics for the follow up of prostate cancer patients.

## Methods

Over a two-month period (Dec 2002–Jan 2003) all the prostate cancer patients being followed up in the Urology outpatient clinics at our institution were audited prospectively. The patients were seen by a Consultant, Specialist Registrar or Senior House Officer. The period of follow-up, initial stage of the disease, management modality, consecutive PSA values and consecutive DRE findings (if available) were recorded on specifically designed data collection forms. All the patients had DRE done on at least one occasion. The change in the management was defined as any alteration in the follow-up pattern; either as an advancement or postponement of a future appointment, the need for further investigation or treatment, the admission of a patient and the referral to a different specialist, for example an Oncologist or Palliative Care specialist

The attending physicians were requested to record whether there was any change in the management and which factors influenced the change. They were specifically requested to record whether DRE influenced a change.

## Results

During the period studied 194 patients being followed up for treated prostate cancer were included. The mean age was 74.8 years and the stages at initial diagnosis were: T1 (n = 73), T2 (n = 63), T3 (n = 44), T4 (n = 14). Ten patients had metastatic disease. The management modalities that these patients had undergone included: hormonal manipulation (68), orchidectomy (8), radical radiotherapy with hormonal manipulation (15), radical radiotherapy (48), radical prostatectomy (21), brachytherapy (1) and active surveillance (33) (Table 1). The management changed in 47 of 194 (24%) patients. The factors that influenced the changes included PSA trend (n = 27), LUTS (n = 10), bone pain (n = 4), change in DRE findings (n = 2) and other factors namely abnormal renal functions (n = 1), hematochezia (n = 1), pruritis (n = 1) and erectile dysfunction (n = 1) (Table 2).

**Table 1:** Management categories of the follow up prostate cancer patients

Management	Number of patients	Percentage of the total number (n = 194)
Active surveillance	33	17
Radical prostatectomy	21	10.8
Radical radiotherapy	48	24.8
Radical radiotherapy With hormones	15	7.8
Brachytherapy	1	0.5
Hormone therapy	68	35
Orchiectomy	8	4.1

**Table 2:** Factors that influenced a change in management

Factors	Number of patients	Percentage of the total number of changes (n = 47)
PSA trend	27	57.5
Lower urinary tract symptoms	10	21.3
Bone pains	4	8.5
DRE findings	2	4.3
Pruritis	1	2.1
Altered renal functions	1	2.1
Erectile dysfunction	1	2.1
Bleeding per rectum	1	2.1

In this audit PSA trend was the most common factor that resulted in a management change. In the two patients there was a change in DRE findings (progression from T_2_b disease to T_3 _disease as observed by the assessor). This only resulted in the subsequent visit being sooner than planned.

## Discussion

The follow up of patients with prostate cancer has traditionally included a disease specific history, serial PSA estimations and a DRE. The roles of PSA and DRE have been extensively evaluated in the diagnosis of prostate cancer patients [9,10]. There have only been a few studies questioning the importance of DRE in the follow up of patients treated with a curative intent [[11-13] and [14]]. These have been based on groups of patients undergoing specific treatments. These studies concluded that DRE is unnecessary in the follow up of patients if PSA is undetectable. However there have been rare case reports describing local or systemic recurrence in the absence of detectable PSA [15,16].

There are no reported studies in the English language assessing the role of routine DRE in the follow up of all treated prostate cancer patients in a general urology outpatient setting. In addition, studies assessing the various factors (e.g LUTS, bone pains etc) that influence a change in the management of these patients have not been reported.

The present audit shows that PSA trend is the most common factor influencing a change in management whilst DRE plays a very limited role. Further, there are other factors that influence a change in the management of these patients' e.g. Bone pain and LUTS.

Although the numbers of patients involved in this audit are moderate it would suggest that Nurse Specialists could deliver the optimum care in following up treated prostate cancer patients. Such Nurse led clinics could be carried out in parallel to the existing Consultant clinics thereby allowing the availability of medical personnel to perform DRE where deemed necessary. A protocol to perform DRE when there is an increase in PSA, onset of new symptoms or worsening of existing symptoms would be suitable for such a clinic. This audit suggests that Nurse Specialists need not be trained to perform DRE before the establishment of such clinics. However larger studies are required to identify subgroups of treated prostate cancer patients who may require a DRE on a regular basis. Alternatively nurses could be taught to undertake DRE thereby further reducing clinician workload. This would require a standardised and validated teaching method, which currently does not exist. In our hospital this audit has influenced the initiation of Nurse led prostate cancer follow up clinics conducted in parallel to the consultant clinics.

## Competing interests

The author(s) declare that they have no competing interests.

## Authors' contributions

NR – Along with VKS conceived the study, collected the data and jointly prepared the text with VKS – Along with NR conceived the study, assessed the data and prepared the text, SG – Participated in collecting patients details and in the preparation of the text, JH – helped in approaching the patients and data collection, SSM – Advised regarding the design of the study and contributed to the text, MEW – Advised regarding the design of the study and contributed to the text, RAB – Overall supervision of the project with periodic assessment on progress and preparation of text

All authors have read and approved the final manuscript.

## Pre-publication history

The pre-publication history for this paper can be accessed here:


